# Serological and morphological prognostic factors in patients with interstitial pneumonia with autoimmune features

**DOI:** 10.1186/s12890-017-0453-z

**Published:** 2017-08-14

**Authors:** Yuhei Ito, Machiko Arita, Shogo Kumagai, Reoto Takei, Maki Noyama, Fumiaki Tokioka, Keisuke Nishimura, Takashi Koyama, Kenji Notohara, Tadashi Ishida

**Affiliations:** 1 Department of Respiratory Medicine, Kurashiki Central Hospital, Kurashiki, Japan; 2 Department of Endocrinology and Rheumatology, Kurashiki Central Hospital, Kurashiki, Japan; 3 Department of Radiology, Kurashiki Central Hospital, Kurashiki, Japan; 4 Department of Pathology, Kurashiki Central Hospital, Kurashiki, Japan

**Keywords:** Interstitial lung disease, Collagen vascular disease, Autoimmune disease, Interstitial pneumonia with autoimmune features, Systemic sclerosis

## Abstract

**Background:**

To identify the prognostic factors for survival in patients with interstitial pneumonia with autoimmune features (IPAF) who meet the serological domain of the IPAF criteria.

**Methods:**

We retrospectively analysed 99 IPAF patients who met the serological domain and were hospitalised at the Respiratory Medicine Unit of Kurashiki Central Hospital from 1999 to 2015. The high-resolution computed tomography findings were usual interstitial pneumonia (UIP; *n* = 1), non-specific interstitial pneumonia (NSIP; *n* = 63), NSIP with organizing pneumonia (OP) overlap (*n* = 15), and OP (*n* = 20). One patient who had radiological UIP pattern, and met the serological and clinical domains was excluded. The clinical characteristics, radiological findings, administered therapy, and prognosis of the remaining 98 IPAF patients who met the serological and morphological domains were analysed.

**Results:**

The median age of the 98 IPAF patients was 68 years, and 41 (41.8%) of them were men. Twelve (12.2%) of the 98 IPAF patients developed other characteristics and were diagnosed with connective tissue disease (CTD) later during the median follow-up of 4.5 years. Univariate Cox analysis revealed systemic sclerosis (SSc)-specific and SSc-associated antibodies (ANA nucleolar pattern, ANA centromere pattern, anti-ribonucleoprotein and anti-Scl-70) positive IPAF, radiological NSIP pattern, bronchoalveolar lavage fluid lymphocytes >15%, and age as significant prognostic factors for survival. Multivariate Cox analysis revealed radiological NSIP pattern (hazard ratio [HR], 4.48; 95% confidence interval [CI], 1.28–15.77, *p* = 0.02) and age (HR, 1.07; 95% CI, 1.02–1.11, *p* = 0.01) were significantly associated with worse survival.

**Conclusions:**

We confirmed that radiological NSIP pattern and age are poor prognostic factors for the survival of IPAF patients. This study suggested that the autoantibodies that are highly specific for certain connective tissue diseases might be less important for the prognosis of IPAF compared with the radiological-pathological patterns. The relatively high proportion of IPAF patients who developed CTD later suggests the importance of careful observation for evolution to CTD in IPAF.

## Background

Many patients with idiopathic interstitial pneumonia (IIP) present clinical features that suggest an underlying autoimmune process but do not meet the established criteria for connective tissue disease (CTD). Interstitial pneumonia with autoimmune features (IPAF) is a term proposed for the condition of such patients.

In IIP patients, survival and prognosis differ according to the histological pattern, baseline pulmonary function, and age [1–4]. However, some questions remain to be addressed. For patients with CTD-associated interstitial lung disease (ILD), the effect of histological pattern on survival is less certain [5–7]. Does the prognosis of IPAF patients differ according to the radiological-pathological pattern? Previous reports suggested that an underlying CTD was important in determining the prognosis of CTD-associated ILD [5]. If IPAF is thought to be a lung-dominant variant of CTD or a CTD preceded by interstitial pneumonia, does the prognosis of IPAF patients differ according to the antibodies that are highly specific for certain CTDs?

The aim of this study was to investigate the prognostic survival factors in IPAF patients who satisfied the serological domain of the IPAF criteria. To answer the 2 aforementioned questions, radiological patterns and specific autoantibodies were included in the analyses.

## Methods

### Study subjects

**Fig. 1:**
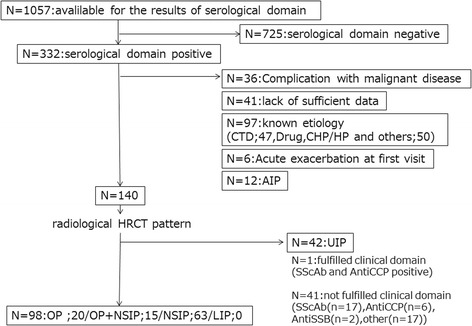
Flow diagram of selection process for patients with interstitial penumonia with autoimmune features who met the serological domein. AIP = acute interstitial penumoniae; Anti-CCP = uclic anti-citrullinated peptide; CTD = connective tissue disease; CHP = Chronic hypersensetivity pneumonitis; HP = hypersensetivity pneumonitis; LIP = lymphocytic interstitial pneumonitis; NSIP + OP = NSIP with OP overlap; NSIP = non-specific interstitial pneumonia; OP = organizing penumonia; SScAb = SSc-specidic and SSc-related antibodies; UIP = usual interstitial pneumonia

We retrospectively screened the medical records of 1057 patients with interstitial pneumonia who were hospitalised at the Respiratory Medicine Unit of Kurashiki Central Hospital from 1999 to 2015. Of these, 332 patients met the serological domain of the IPAF criteria. We excluded 192 patients due to insufficient data (*n* = 41), complication with malignant disease at first visit (*n* = 36), known causes (CTD, *n* = 47; drug, chronic hypersensitivity, pneumonitis, and others, *n* = 50), acute interstitial pneumonia (*n* = 12), and acute exacerbation at first visit (*n* = 6). CTD was diagnosed when the patients fulfilled the American College of Rheumatology criteria [8–13]. High-resolution computed tomography (HRCT) images were classified as definite usual interstitial pnaumonia (UIP) pattern, possible UIP pattern, or inconsistent with UIP pattern according to the guidelines for idiopathic pulmonary fibrosis (IPF) [14]. Definite UIP and possible UIP pattern were defined as UIP pattern in this study. The cases interpreted as inconsistent with UIP pattern were further classified as nonspecific interstitial pneumonia (NSIP) pattern, NSIP with organizing pneumonia (OP) overlap, or OP pattern according to the American Thoracic Society (ATS)/European Respiratory Society (ERS) statement of IIP 2013 [15] and IPAF 2015 [16]. HRCT findings of NSIP were defined as basal predominant reticular abnormalities with traction bronchiectasis, peri-bronchovascular extension and subpleural sparing, frequently associated with ground-glass attenuation. HRCT findings of OP were defined as bilateral patchy areas of consolidation with a subpleural and lower lung zone predominance. NSIP with OP was defined as basal predominant consolidation, often peri-diaphragmatic, associated with features of fibrosis. HRCT images of remaining 140 patients were classified as UIP in 42, NSIP in 63, NSIP with OP in 15, OP in 20 patients based on the predominant pattern. Among 42 patients with UIP pattern, only 1 fulfilled the clinical domain (Raynaud’s phenomenon); therefore, in this analysis, we excluded this 1 patient who satisfied serological and clinical domains, considering the rarity of this entity, and focused on the remaining 98 patients with IPAF who satisfied serological and morphological domains (Fig. 1). All patients were carefully examined by rheumatologists and the absence of CTD was confirmed. HRCT was performed with 1.0-mm sections. HRCT scans at first visit were randomised and reviewed by 2 expert pulmonologists of 30 years’ and 5 years’ experience (MA, YI) and one expert radiologist of 24 years’ experience (TK). All disagreements were resolved through consensus.

### Data collection

Clinical data were obtained retrospectively from patient records. We evaluated patients’ characteristics, pulmonary function tests, bronchoalveolar lavage (BAL), and serological test results. These tests were conducted for all patients within 1 month of the first diagnosis of interstitial pneumonia. Spirometry and the lung’s diffusing capacity for carbon monoxide (DLCO) were measured according to the ATS/ERS recommendation as physiological assessments and were examined within 1 month of the first diagnosis of interstitial pneumonia [17, 18]. Abnormal cell counts in BAL fluid were neutrophils >3%, lymphocytes >15%, and eosinophils >1% [19]. Response to treatment was evaluated according to the official ATS/ERS/JRS/ALAT Statement of Idiopathic Pulmonary Fibrosis 2011 [14]. Deterioration was defined as a condition in which a > 5–10% decline in FVC and >15% decline in DLCO occurred over 6 or 12 months.

### Specific antibodies

ANA nucleolar pattern, ANA centromere pattern, anti-ribonucleoprotein, anti-Scl-70, anti-cyclic citrullinated peptide (CCP), anti-Jo1 antibody (Jo1), anti-tRNA synthetase (anti-tRS) antibodies other than anti-Jo1 (ARS other than anti-Jo1), and anti-La (SSB) were included as CTD-specific antibodies. ANA nucleolar pattern, ANA centromere pattern, anti-ribonucleoprotein, and anti-Scl-70 were defined as systemic sclerosis (SSc)-specific and SSc-related antibodies (SScAb). All patients were subdivided into 5 groups, SScAb, anti-CCP, ARS Ab, anti-SSB positive IPAF, and other IPAF, according to the specific antibodies. Seven patients were positive with more than 2 CTD-specific antibodies (SScAb and anti-CCP were present in 1 patient; SScAb and anti-Jo1 in 2; SScAb and anti-SSB in 1; SScAb and anti-SSB in 1; anti-SSB and anti-Jo1 in 1; and SScAb, anti-SSB, and anti-CCP in 1). These 7 patients and those who tested negative for all specific antibodies were grouped under other IPAF.

### Histological assessment

Among the 98 IPAF patients, 17 underwent surgical lung biopsies (SLB). The major histological patterns were classified according to the current IIPs classification 2013 [15]. Interstitial lymphoid aggregates with germinal centres and diffuse lymphoplasmacytic infiltration were evaluated [16].

### Statistical analyses

Continuous variables are presented as the median and interquartile range. Categorical variables are described as counts and percentages. To detect differences in three groups, we used Kruskal-Wallis test for continuous variables and Steel-Dwass test for post hoc analysis and Fisher’s exact test for categorical variables. Survival was evaluated using the Kaplan-Meier survival curves and log-rank test. Cox proportional hazards regression analysis was used to identify significant variables for predicting survival status. Variables selected via the univariate test (*p* < 0.05) were evaluated using multivariate Cox regression analysis. A *p* value <0.05 was considered statistically significant.

## Results

### Clinical, radiological, and physiological characteristics of study participants stratified according to HRCT

Of the 98 IPAF patients, 20 (20.4%) were radiologically classified as OP, 15 (15.3%) as NSIP + OP, and 63 (64.3%) as NSIP. The clinical characteristics stratified according to HRCT are shown in Table 1. The proportion of subacute onset was higher in OP and NSIP + OP patterns than in NSIP pattern. BAL fluid data at first presentation were available in 75 (76.5%) IPAF patients. The percentage of BAL fluid lymphocytes was lower in those with NSIP pattern than in those with OP (*p* = 0.001, Steel-Dwass test) and NSIP + OP patterns (*p* = 0.013, Steel-Dwass test). C-reactive protein (CRP) was higher in those with OP pattern than those with NSIP + OP (*p* = 0.004, Steel-Dwass test) and NSIP pattern (*p* = 0.006, Steel-Dwass test). KL6 was lower in those with OP pattern than in those with NSIP + OP (*p* < 0.001, Steel-Dwass test) and NSIP pattern (*p* < 0.001, Steel-Dwass test).

**Table 1:** Clinical, radiographic and physiologic characteristics of study participants stratified by hrct pattern

		HRCT pattern			
	total	OP	NSIP + OP	NSIP	*p* value
Number of patients, n	98	20 (20.4%)	15 (15.3%)	63 (64.3%)	
Age, yr	67.5 [59.0, 76.0]	68.5 [64.8, 73.8]	67.0 [52.5, 76.0]	68.0 [59.0, 76.0]	0.88
Male gender, n (%)	41 (41.8%)	10 (50.0%)	6 (40.0%)	25 (39.7%)	0.693
Smoking					
never smokers, n	60 (61.2%)	12 (60%)	11 (73.3%)	37 (58.7%)	0.653
former/current smoker, n	38 (38.8%)	8 (40%)	4 (26.7%)	26 (41.3%)	
Onset					
subacute, n (%)	36 (36.3%)	20 (100%)	14 (93.3%)	2 (3.2%)	<0.001
chronic, n (%)	62 (63.3%)	0(0%)	1 (6.7%)	61 (96.8%)	
Laboratory data					
WBC,103/m m^3^	6900 [5725, 900]	775 [6500, 10,325]	8900 [6000, 9700]	6500 [5600, 7650]	0.03
CRP,g/dL	0.46 [0.14, 1.92]	7.60 [3.25, 11.59]	1.03 [0.50, 1.81]	0.25 [0.11, 0.59]	<0.001
KL6,U/ml	945 [552, 1673]	321[280, 337]	1172 [887, 3049]	1135 [771, 1775]	<0.001
Pulmonary function %predicted	*n* = 85	*n* = 11	*n* = 13	*n* = 61	
FVC, %predicted	76.3 [62.7, 88.6]	81.2 [72.1, 87.9]	50.9 [42.1, 65.2]	54.8 [46.2, 71.9]	0.2
DLCO, %predicted	56.6 [46.4, 76.3]	82.9 [75.6, 100.6]	67.9 [57.1, 87.1]	75.6 [62.7, 88.0]	0.009
BAL fluid	*n* = 75	*n* = 16	*n* = 12	*n* = 47	
Lymphocytes, %	19.0 [10.0,39.0]	53.0 [27.5, 71.3]	34.9 [16.2, 48.5]	13.0 [8.5, 24.1]	<0.001
Neutrophils, %	6.0 [1.9,12.5]	7.5 [1.8, 14.6]	9.7 [5.1, 16.3]	4.0 [1.2, 10.5]	0.225
Eosinophils, %	1.7 [0.9,4.7]	2.0 [1.0, 12.0]	3.7 [1.4, 5.0]	1.0 [0.3, 3.3]	0.177
Autoantibodies, n					
SSc-specific and SSc-reralted antibodies, n	36	3	2	31	
Nucleolar-ANA, n	15	0	0	15	
Anti-Centromere, n	4	1	0	3	
Anti-RNP, n	9	2	1	6	
Anti-Scl-70, n	8	0	1	7	
Anti-CCP, n	15	3	2	10	
Anti-Jo1, n	9	2	3	4	
ARS Ab other than anti-Jo1, n	4	0	3	1	
Anti-La/SSB, n	7	1	4	3	
ANA (≧1:320), n	28	1	4	23	
Rheumatoid factor (>60 IU/ml), n	28	8	3	17	
Anti-Ro/SSA, n	18	6	3	9	
Anti-dsDNA, n	6	1	2	3	
Anri-Sm, n	4	1	0	3	

Some patients had multiple serological tests. Data are presented as No. (%), median(range). *ANA* antinuclear antibody, *Anti-CCP* uclic anti-citrullinated peptide, *Anti-dsDNA* anti-double stranded DNA, *Anti-RNP* anti ribonucleoprotein, *ARS Ab* anti-tRNA synthetase antibodies, *Anti-Scl-70* anti-Sclero 70, *BAL* bronchoalveolar lavage, *CRP* C reactive protein, *DLCO* diffusing capacity of the lung for carbon monoxide, *FVC* forced vital capacity, *IPAF* Interstitial pneumonia with autoimmune features, *NSIP* non-specific interstitial pneumonia, *NSIP + OP* NSIP with OP overlap, *OP* organizing penumonia, *SScAb* SSc-related antibody

### Therapy and prognosis of study participants stratified according to HRCT

Therapy and prognosis of study participants stratified according to HRCT patterns are shown in Table 2. Treatment for interstitial pneumonia was introduced in 78 (79.6%) patients. The 5-year survival rates were 100%, 86.7%, and 58.6% for OP, NSIP + OP, and NSIP, respectively.

**Table 2:** Therapy and prognosis of study participants stratified by HRCT pattern

			HRCT pattern					
	Total		OP		NSIP + OP		NSIP	
Number of patients, n	98		20		15		63	
5-year survival, %	71.1%		100.0%		86.7%		58.6%	
Treatment,n (%)	78	(79.6%)	19	(95%)	15	(100%)	44	(69.8%)
corticosteroid, n(%)	17	(17.3%)	15	(75%)	6	(40%)	6	(9.5%)
corticosteroid + IS^a,^ n (%)	48	(49.0%)	4	(20%)	9	(60%)	35	(55.6%)
corticosteroid + IS + pirfenidone, n (%)	1	(1.0%)	0	(0%)	0	(0%)	1	(1.6%)
pirfenidone, n (%)	2	(2.0%)	0	(0%)	0	(0%)	2	(3.2%)
Response to Treatment, n (%)	70	(71.4%)	19	(95%)	15	(100%)	36	(57.1%)
Improve/stable/deteriorate, n	45/20/5		18/1/0		14/1/0		13/18/5	
Overall death, n (%)	27	(27.6%)	1	(5%)	3	(20%)	23	(36.5%)
Death due to respiratory failure, n (%)	15	(15.3%)	0	(0%)	1	(6.7%)	14	(22.2%)
Acute exacerbation, n (%)	5	(5.1%)	0	(0%)	0	(0%)	5	(7.9%)

Data are presented as No. (%). *NSIP* non-specific interstitial pneumonia, *NSIP + OP* NSIP with OP overlap, *OP* organizing penumonia. ^a^IS = immnuno suppressants other than corticosteroid which included azathioprine, cyclsporin, cyclophosphamide and tacrolimus

### HRCT pattern, administered therapy, and prognosis of study participants stratified according to specific antibodies

HRCT patterns stratified according to the specific antibodies are shown in Table 3. The NSIP pattern was relatively common among patients with SScAb and anti-CCP-positive IPAF. The 5-year survival rates were 42.1%, 66.7%, 75.8%, 100.0%, and 75.8% in SScAb-positive, anti-CCP-positive, ARS Ab-positive, Anti-SSB-positive, and other IPAF, respectively.

**Table 3:** HRCT pattern, therapy and prognosis of study participants stratified by specific antibodies

			Specicifc antibodies									
	Total		SScAb positive IPAF		Anti-CCP positive IPAF		ARS Ab positive IPAF		Anti-SSB positive IPAF		Other IPAF	
Number of patients, n	98		28		13		9		4		44	
HRCT pattern												
OP, n (%)	20	(20.4%)	3	(10.7%)	3	(23.1%)	2	(22.2%)	1	(25%)	11	(23.4%)
NSIP + OP, n (%)	15	(15.3%)	1	(3.6%)	1	(7.7%)	4	(44.4%)	2	(50%)	7	(14.9%)
NSIP, n (%)	63	(64.3%)	24	(85.7%)	9	(69.2%)	3	(33.3%)	1	(25%)	26	(55.3%)
5-year survival,%	71.1%		42.1%		66.7%		75.8%		100.0%		75.8%	
Treatment,n (%)	79	(80.6%)	23	(82.1%)	10	(76.9%)	7	(77.8%)	4	(100%)	34	(77.2%)
corticosteroid, n (%)	27	(27.6%)	5	(17.9%)	2	(15.4%)	2	(22.2%)	3	(75%)	15	(34.1%)
corticosteroid + IS^a^, n (%)	44	(44.9%)	16	(57.1%)	6	(46.2%)	5	(55.6%)	1	(25%)	16	(36.4%)
corticosteroid + IS + pirfenidone, n (%)	1	(1.0%)	0	(0%)	0	(0%)	0	(0%)	0	(0%)	1	(2.3%)
IS only, n (%)	5	(5.1%)	2	(7.1%)	2	(15.4%)	0	(0%)	0	(0%)	0	(0%)
pirfenidone, n (%)	2	(2.0%)	0	(0%)	0	(0%)	0	(0%)	0	(0%)	2	(4.5%)
Response to Treatment, n (%)	70	(71.4%)	20	(71.4%)	9	(69.2%)	7	(77.8%)	4	(100%)	30	(68.1%)
Improve/stable/deteriorate, n	44/20/6		7/11/2		5/3/1		5/1/1		4/0/0		23/5/2	
Overall death, n (%)	27	(27.6%)	13	(46.4%)	3	(23.1%)	1	(11.1%)	0	(0%)	10	(22.7%)
Death due to respiratory failure,n (%)	15	(15.3%)	8	(28.6%)	1	(7.7%)	0	(0%)	0	(0%)	6	(13.6%)
Acute exacerbation, n (%)	5	(5.1%)	2	(7.1%)	0	(0%)	0	(0%)	0	(0%)	3	(6.8%)

Data are presented as No.(%). *Anti-CCP* uclic anti-citrullinated peptide, *ARS Ab* anti-tRNA synthetase antibodies, *IPAF* Interstitial pneumonia with autoimmune features, *IS* immnuno suppressants other than corticosteroid, *NSIP* non-specific interstitial pneumonia, *NSIP + OP* NSIP with OP overlap, *OP* organizing penumonia, *SScAb* SSc-specific and SSc-related antibodies.^a^IS = immnuno suppressants other than corticosteroid which included azathioprine, cyclsporin, cyclophosphamide and tacrolimus

### Survival and prognostic factors

**Fig. 2:**
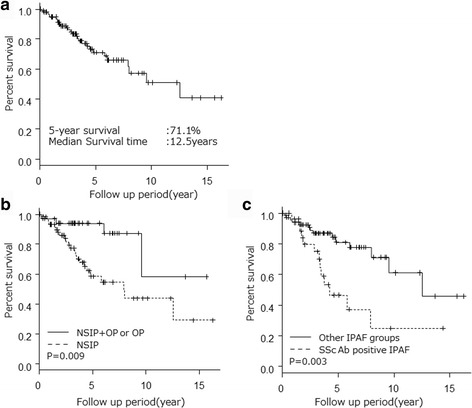
Comparison of the survival curves of patients with IPAF. **a** Survival of patients with IPAF; **b** Survival of patients with NSIP + OP or OP and NSIP; **c** Survival of patients with SScAb-positive IPAF and the other IPAF groups. The other IPAF goups included patients with Anti-CCP positive, ARS Ab positive, Anti-SSB positive IPAF and other IPAF. Anti-CCP = anti-cyclic citrullinated peptide; ARS Ab = anti-tRNA synthetase antibodies; IPAF = Interstitial pneumonia with autoimmune features; NSIP = non-specific interstitial pneumonia; OP = organizing pneumonia; NSIP + OP = NSIP with OP overlap; SscAb = SSc-related antibody

Twenty-seven patients (27.6%) died during the median follow-up period of 4.58 years. The causes of death were documented as respiratory failure (*n* = 15), lung cancer (*n* = 3), other malignant disease (*n* = 3), severe infection (*n* = 2), acute myocardial infarction (*n* = 1), gastrointestinal perforation (*n* = 1), and unknown (*n* = 2). Five patients died due to acute exacerbation amoung 15 patients who died due to respiratory failure. In patients with IPAF, the 5-year survival was 71.1% and median survival time was 12.5 years. Patients with NSIP pattern had significantly worse survival than those with NSIP + OP or OP patterns (*p* = 0.009). Patients with SSAb-positive IPAF had significantly worse survival than those with other IPAF groups (*p* = 0.003) (Fig. 2). Univariate Cox analysis revealed SScAb-positive IPAF (hazard ratio [HR], 2.88; 95% CI, 1.36–6.11; *p* = 0.01), radiological NSIP pattern (HR, 4.00; 95% CI, 1.38–11.6, *p* = 0.01), BAL fluid lymphocytes >15% (HR, 0.29; 95% CI, 0.103–0.815, *p* = 0.02), and age (HR, 1.07; 95% CI, 1.03–1.11, *p* < 0.01) to be significant prognostic survival factors (Table 4). Multivariate Cox analysis revealed radiological NSIP pattern (HR, 4.48; 95% CI, 1.28–15.77, *p* = 0.02) and age (HR, 1.07; 95% CI, 1.02–1.11, *p* = 0.01) to be poor prognostic factors for survival (Table 5).

**Table 4:** Prognostic survival factors in patients with IPAF using univariate cox model

	Number	HR	[95% CI]	*p* value
Age	98	1.07	[1.03–1.11]	<0.01
Male sex	98	1.98	[0.92–4.25]	0.08
Smoker	98	1.27	[0.59–2.73]	0.54
Radiologic NSIP pattern vs. NSIP + OP or OP pattern	98	4.00	[1.38–11.6]	0.01
SScAb positive IPAF	98	2.88	[1.36–6.11]	0.01
Anti-CCP positive IPAF	98	0.87	[0.26–2.91]	0.82
Anti-SSB positive IPAF	98	NE		1.00
ARS Ab positive IPAF	98	0.25	[0.03–1.89]	0.18
BAL fluid Lymphocytes > 15%	75	0.29	[0.10–0.82]	0.02
BAL fluid neutrophil > 3%	75	1.99	[0.65–6.06]	0.23
BAL fluid eosinophil > 1%	75	0.63	[0.25–1.59]	0.33
FVC%predicted	85	0.98	[0.96–1.00]	0.08
DLCO%predicted	85	0.99	[0.97–1.02]	0.55

*Anti-CCP* uclic anti-citrullinated peptide, *ARS Ab* anti-tRS antibody, *BAL* bronchoalveolar lavage, *CI* confidence interval, *DLCO* diffusing capacity of the lung for carbon monoxide, *FVC* forced vital capacity, *IPAF* Interstitial pneumonia with autoimmune features, *NE* not evaluable, *NSIP* non-specific interstitial pneumonia, *NSIP + OP* NSIP with OP overlap, *OP* organizing penumonia, *SScAb* SSc-specific and SSc-related antibodies

**Table 5:** Prognostic survival factors in patients with IPAF using multivariate cox model

	n	HR	[95% CI]	*p* value
Radiologic NSIP pattern vs. NSIP + OP or OP pattern	98	4.48	[1.28–15.77]	0.02
Age	98	1.07	[1.02–1.12]	0.01

*CI* confidence interval, *NSIP* non-specific interstitial pneumonia, *NSIP + OP* NSIP with OP overlap, *OP* organizing penumonia

### Histopathological findings, characteristics, and outcomes of IPAF patients who underwent surgical lung biopsy

Seventeen patients (17.3%) underwent SLB; 13 (20.6%) with radiological NSIP, 3 (20.0%) with NSIP + OP, and 1 (5%) with OP pattern. Interstitial lymphoid aggregates with germinal centres were observed in 9 (52.9%) patients, diffuse lymphoplasmacytic infiltration in 13 (76.5%), and either of those features in 14 (82.4%). Of the 13 patients presenting a radiological NSIP pattern, pathological diagnosis was NSIP in 8, UIP in 3, and unclassifiable in 2 patients. Of the 3 patients presenting a radiological NSIP + OP pattern, pathological diagnosis was also an NSIP + OP in 2, and NSIP in 1 patient. One patient presenting a radiological OP pattern also showed a OP pattern pathologically. Three patients presenting a radiological NSIP pattern and a pathological UIP pattern had relatively poor prognosis; two died due to respiratory failure during the follow-up period (34.5 and 30.5 months from the first visit respectively). On the other hand, amoung 8 patients with concordant NSIP pattern (i.e., radiological NSIP and a pathological NSIP pattern), 2 died during the follow-up period, and the median survival was 95.7 months (95% CI, 69.8 months- not reached).

### Progression to CTD

Twelve (12.2%) of the 98 IPAF patients developed other characteristics and were diagnosed with CTD (rheumatoid arthritis (RA), *n* = 7; systemic sclerosis, *n* = 2; systemic lupus erythematosus, *n* = 1; Sjogren’s syndrome and systemic sclerosis, *n* = 1; and dermatomyositis and systemic sclerosis, *n* = 1) later during the median follow-up of 4.5 years (range: 1.88–6.07 years; Table 6). Six patients (9.5%) presenting with a radiological NSIP pattern were diagnosed with CTD later. Two patients (13.3%) presenting with an NSIP + OP pattern were diagnosed with CTD later. Four patients (20%) presenting with an OP pattern were diagnosed with CTD later.

**Table 6:** Characteristics of patients with IPAF who progressed to connective tissue disease

Number	CTD	Duration (month)	Specific antibody	HRCT pattern
1	RA	62.2	Anti-RNP, anti-CCP	NSIP + OP
2	SS + SSc	88.2	Anti-La/SSB	NSIP + OP
3	RA	10.7	Anti-CCP	OP
4	RA	3.4	Anti-CCP	OP
5	RA	20.3	Anti-CCP	OP
6	RA	24.5	Anti-CCP	NSIP
7	RA	32.8	Anti-CCP	NSIP
8	SSc + DM	29.5	Anti-RNP	NSIP
9	SSc	60.1	Anti-Scl-70	NSIP
10	SLE	26.3	not detected	OP
11	SSc	56.4	not detected	NSIP
12	RA	130.3	not detected	NSIP

*Anti-CCP* anti-cyclic citrullinated peptide, *Anti-RNP* anti ribonucleoprotein, *Anti-Scl-70* anti-Sclero 70, *CTD* connective tissue disease, *DM* dermatomyositis, *NSIP* non-specific interstitial pneumonia, *NSIP + OP* NSIP with OP overlap, *OP* organizing penumonia, *RA* Rheumatoid arthritis, *SLE* systemic lupus erythematosus, *SS* sjogren syndrome, *SSc* Systemic sclerosis

## Discussion

We confirmed here that radiological NSIP pattern and age are poor prognostic factors for survival in patients with IPAF patients who meet serological and morphological domain.

Univariate Cox analysis revealed radiological NSIP pattern, age, BAL fluid lymphocytes >15%, and SScAb-positive IPAF to be significant prognostic survival factors. First, the fact that radiological NSIP pattern is a poor prognostic factor for survival has been previously reported in cases of patients with IIP wherein the prognosis of patients with cryptogenic organizing pneumonia (COP) was better than those with NSIP. Nagai et al. compared the prognosis of 31 patients with idiopathic NSIP (iNSIP) with 16 COP patients. While no COP patient died or worsened, 2 iNSIP patients died and 3 worsened. They concluded that the prognosis of COP patients was better than that of iNSIP patients [3]. In this study, 13 (20.6%) patients with radiological NSIP pattern underwent surgical lung biopsy, and 3 showed UIP pattern pathologically. In patients with IIP and CTD-ILD, the discordance between radiological and pathological diagnosis is also reported in previous studies. Patients with concordant UIP had the highest mortality, while concordant NSIP had the lowest mortality. Discordant NSIP (i.e., radiological diagnosis of UIP or indeterminate, with histological diagnosis of NSIP) and discordant UIP (i.e., radiological diagnosis of NSIP or indeterminate, with histological diagnosis of UIP) were associated with lower mortality than those with concordant UIP, but greater mortality than those with concordant NSIP [20, 21]. Three patients with discordant UIP may have influenced our result that radiological NSIP pattern had poor prognosis compared with NSIP + OP and OP pattern. In patients with radiological NSIP pattern, surgical lung biopsy should be considered to predict prognosis accurately, if possible. Furthermore, in the current study, differences and trends in disease onset, CRP, BAL findings, and KL6 among IPAF patients stratified according to HRCT were similar to those with IIPs, suggesting a similarity in the characteristics of IPAF and IIPs. Second, SScAb-positive IPAF was a significant poor prognostic factor for survival. This poor prognosis of patients with SScAb-positive IPAF might be mainly due to the lower frequency of good prognostic OP pattern. Among patients with SScAb-positive IPAF, good prognostic OP pattern was seen only in 3 (12%) patients. In this study, anti-CCP-positive IPAF was not a significant prognostic survival factor. In contrast to other types of CTD, the reported prognosis of RA-ILD was relatively worse [22–24]. Park et al. reported that the survival of RA-ILD patients was not significantly worse than that of patients with other-non RA-CTD-ILD. However, using Kaplan-Meier survival analysis, they also reported that the survival of RA-UIP patients was similar to that of IPF/UIP patients and significantly worse than that of non-RA-CTD-UIP patients [6]. As these reports suggested, in RA patients, UIP pattern is often identified and thought to be a poor prognostic factor. In this study, although UIP pattern was often identified in patients with anti-CCP-positive IIP, all these patients could not be diagnosed with IPAF and thus were excluded. This might be partly the reason why anti-CCP-positive IIP was not a prognostic survival factor. Third, BAL fluid lymphocytes >15% was a significant favourable prognostic survival factor. Patients with BAL lymphocytes >15% might have a favourable prognostic pathological pattern. In a previous report, the average BAL fluid lymphocytes count was 37.3%, 44%, and 7.2% in patients with iNSIP, COP, and IPF, respectively [3]. Another study reported that the average BAL fluid lymphocytes count was 40.5%, 19%, and 5.5% in patients with cellular NSIP, fibrotic NSIP, and IPF. BAL fluid lymphocytosis was a significant favourable prognostic factor in those with fibrotic interstitial pneumonia [25].

Multivariate Cox analysis revealed radiological NSIP pattern and age as independent poor prognostic survival factors. Testing positive for SScAb was a significant poor prognostic survival factor in univariate analysis, but not in multivariate analysis. This suggested that compared to radiological-pathological patterns the autoantibodies that are highly specific for certain CTDs are less important in the prognosis of IPAF. Since CTD is a systemic disease, the prognosis of CTD-ILD may also be affected by organ dysfunctions other than ILD. For example, pulmonary arterial hypertension, chronic kidney disease and interstitial lung disease were reported to be the poor prognostic survival factors of SSc patients [26]. On the other hand, lung manifestation was usually the sole organ dysfunction in IPAF patients, and the prognosis might be more strongly affected by lung manifestation itself than the other organ dysfunction associated with each specific antibodies. In this study, age is also an independent poor prognostic survival factor independent of HRCT pattern. This result might have just represented life expectancy unrelated to the interstitial lung disease iteself as older patients have a shorter expected life-span.

In this analysis, 3 important findings suggested that IPAF was a lung-dominant variant of CTD or a CTD preceded by interstitial pneumonia. First, 12 (12.2%) of the 98 IPAF patients later developed CTD. Fischer et al. reported that 3 of 74 patients who were positive for anti-CCP but not RA-ILD later developed RA [27]. Lee et al. reported that 3 of 18 biopsy-proven RA-ILD patients were not diagnosed with RA at first but later developed RA [6]. Bauer et al. reported that among 19 SSc-ILD patients, ILD occurred usually after the diagnosis of SSc in 16 patients but ILD occurred beforehand in 3 patients [26]. These reports suggest that some patients presented with ILD before being diagnosed with CTD. Pereira DA et al. reported that amoung 52 patients with lung-dominant CTD, 8 developed CTD later during the median follow up of 48 months [28]. In contrast, Chartrand et al. reported that amoung 56 patients with IPAF, none developed CTD later during the median follow up of 284.9 weeks [29]. One possible reason for these differences might be the diversity of the proportion of suggested CTD according to the diagnostic criteria in each cohort (e.g. positive rates of specific antibodies corresponding to each CTD may be different in each cohort). Further studies are needed to investigate this issue. However, the relatively high proportion of IPAF patients who developed CTD later in this study suggests the importance of longitudinal surveillance for evolution to CTD among those with IPAF and those with IIP in general. Second, of the 17 patients who underwent SLB, interstitial lymphoid aggregates with germinal centres and diffuse lymphoplasmacytic infiltration were frequently seen. These 2 histological findings are considered to be highly associated with CTD [16]. Omote et al. reported that among 44 patients with serologically positive lung-dominant CTD, interstitial lymphoid aggregates with germinal centres were observed in 21 patients (48%) and diffuse lymphoplasmacytic infiltration was observed in 32 (73%) [30]. Song et al. reported that cases of IPF/UIP with positive autoantibodies had more characteristic histological features, such as germinal centres and plasma cells than did those of IPF/UIP without autoantibodies [31]. Third, HRCT patterns of IPAF patients stratified according to specific antibodies were similar to those of corresponding CTDs. In this study, patients with SScAb-positive IPAF showed a higher frequency of NSIP pattern than OP pattern. Previous reports showed that in patients with SSc, NSIP was frequently seen while OP was rare pathologically [32–34].

When we found patients with interstitial pneumonia to be positive with specific antibodies suggestive of an underlying CTD, we first administered immunosuppressive therapy. It is very important to identify the underlying cause in patients with ILD before diagnosing them with IIP as underlying causes help determine treatment principles. As this study and previous studies have shown, the IPAF criteria might be useful in identifying ILD patients suspected to have an underlying CTD. It is also reasonable to treat those IPAF patients with immunosuppressive treatments that are effective for certain suspected CTDs according to the specific antibodies.

This study has some limitations. First, we investigated only IPAF patients who satisfied the serological and morphological domains of the IPAF criteria. We did not investigate those who met the clinical and morphological domains. We excluded one patient who met the serological and clinical domains, although this subset seemed to be comparatively rare. Second, this was a retrospective single-centre study with a small sample size.

## Conclusions

We confirmed that radiological NSIP pattern and age are significant poor prognostic factors for the survival of IPAF patients. This study suggested that the autoantibodies that are highly specific for certain CTDs might be less important for the prognosis of IPAF compared with the radiological-pathological patterns. The relatively high proportion of IPAF patients who developed CTD later suggests the importance of careful observation for evolution to CTD in IPAF.

## Abbreviations

anti-RA: anti-tRNA synthetase
ATS: American Thoracic Society
BAL: bronchoalveolar lavage
CCP: anti-cyclic citrullinated peptide
CI: confidence interval
CTD: connective tissue disease
DLCO: diffusing capacity for carbon monoxide
ERS: European Respiratory Society
HR: hazard ratio
HRCT: high-resolution computed tomography
IIP: idiopathic interstitial pneumonia
ILD: nterstitial lung disease
iNSIP: idiopathic NSIP
IPAF: Interstitial pneumonia with autoimmune features
IPF: idiopathic pulmonary fibrosis
Jo1: anti-Jo1 antibody
NSIP: non-specific interstitial pneumonia
OP: organizing pneumonia
RA: rheumatoid arthritis
SLB: surgical lung biopsy
SSc: systemic sclerosis
SScAb: SSc-specific and SSc-associated antibodies
UIP: usual interstitial pneumonia
